# Supplementary material for: A gentle tutorial on accelerated parameter and confidence interval estimation for hidden Markov models using Template Model Builder
Source: Biom J. 2022 May 27;64(7):1260–88. doi: 10.1002/bimj.202100256 (PMC9796807; doi:10.1002/bimj.202100256)
Supplement: Supplementary file 1 — Supporting Information for this article is available from the authors or on the WWW under https://timothee‐bacri.github.io/HMM_with_TMB [file BIMJ-64-1260-s001.zip › README.pdf]

README

Reproducing analysis

- Open *hmm-tmb.Rproj*, or setup the working directory to the main folder manually.
- Go in the folder *code/*.
- Open the file *main.R*.
- Change the variables `RUN_LAMB`, `RUN_SIMULATION1`, `RUN_SIMULATION2`, `RUN_SIMULATION3`, `RUN_SIMULATION4`, and `RUN_TINNITUS` to `TRUE` depending on which dataset analysis you are interested in reproducing.
- We advise adjusting advanced settings in *code/setup_parameters.R* and reduce drastically the number of coverage, bootstrap, and benchmark samples (the corresponding variables are respectively `COVERAGE_SAMPLES`, `BOOTSTRAP_SAMPLES`, and `BENCHMARK_SAMPLES` (along with `CONSISTENCY_BENCHMARK_TINN` in the case of the tinnitus dataset)) to 2 or 3 and increase if your computer can handle it in a reasonable time. These variables can be set to 0.
- Execute `source("code/main.R")`.

Be aware that the analysis with default settings takes about 6 days on a Linux computer with a good processor and 32 threads. More details at https://timothee-bacri.github.io/HMM_with_TMB/github.html#poi_hmm_tinn.R.

Loading data

- Execute `source("code/main.R")`.

Reproducing figures + tables + results

All figures and tables present in the article *paper.pdf* are produced in the file *paper.rnw* in the same folder as this *README.pdf* file. These code chunks can be run after loading the data as detailed above. Each code chunk producing a table of figure is preceded in *paper.rnw* by a comment such as `Table 5` or `Figure 2`, to make searching for these code chunks as easy as possible. With RStudio, code chunks can be run by either

- using the shortcut `Ctrl+Shift+Enter`, or
- copy-pasting their code in the console and pressing `Enter`, or
- clicking on the downward arrow near the `Run` button at the top-right corner of the code window, then selecting `Run current chunk`.

To generate the pdf of the article along with its results and figures and tables, please install the R package `knitr` then execute the command

```
knitr::knit2pdf("paper.rnw")
```

Note that you need to have a TeX distribution installed and setup. If you don't have one, you can install `tinytex` via the 2 commands

```
install.packages("tinytex")
tinytex::install_tinytex()
```

then you can knit the article.

Be aware that knitting *paper.rnw* (or this current file) will execute *code/main.R*, and will run the analysis instead of loading it if one sets some of the variables `RUN_LAMB`, `RUN_SIMULATION1`, `RUN_SIMULATION2`, and `RUN_TINNITUS` defined to `TRUE` in *code/main.R*. Also note that executing that file will execute in turn *code/setup_parameters.R*, which will compile necessary `TMB` files to generate dynamic libraries (files in the *code/* folder, ending with `.dll` and `.o`) if they are not already present. This process takes a few minutes, but only needs to be done once. Re-executing the scripts or re-knitting will be quick after the first time. Note that these dynamic libraries are specific to the operating system. For example, the libraries compiled on Windows will not work on Linux operating systems.

Software version information

The following was executed on the computer that ran the simulation study.

```
> sessionInfo()
R version 3.6.3 (2020-02-29)
Platform: x86_64-pc-linux-gnu (64-bit)
Running under: Ubuntu 18.04.6 LTS

Matrix products: default
BLAS:   /usr/lib/x86_64-linux-gnu/atlas/libblas.so.3.10.3
LAPACK: /usr/lib/x86_64-linux-gnu/atlas/liblapack.so.3.10.3

locale:
 [1] LC_CTYPE=en_DK.UTF-8       LC_NUMERIC=C
 [3] LC_TIME=en_DK.UTF-8        LC_COLLATE=en_DK.UTF-8
 [5] LC_MONETARY=en_DK.UTF-8    LC_MESSAGES=en_DK.UTF-8
 [7] LC_PAPER=en_DK.UTF-8       LC_NAME=C
 [9] LC_ADDRESS=C               LC_TELEPHONE=C
[11] LC_MEASUREMENT=en_DK.UTF-8 LC_IDENTIFICATION=C

attached base packages:
[1] parallel  stats     graphics  grDevices utils     datasets  methods
[8] base

other attached packages:
 [1] mvtnorm_1.1-3        doParallel_1.0.16    iterators_1.0.13
 [4] foreach_1.5.2        ggpubr_0.4.0         ggplot2_3.3.5
 [7] R.utils_2.11.0       R.oo_1.24.0          R.methodsS3_1.8.1
[10] xtable_1.8-4         knitr_1.37           ggthemes_4.2.4
[13] microbenchmark_1.4.9 TMB_1.7.22

loaded via a namespace (and not attached):
 [1] pillar_1.6.4      compiler_3.6.3   tools_3.6.3      lifecycle_1.0.1
 [5] tibble_3.1.6      gtable_0.3.0     lattice_0.20-45  pkgconfig_2.0.3
 [9] rlang_0.4.12      Matrix_1.4-0     DBI_1.1.2        xfun_0.29
[13] withr_2.4.3       dplyr_1.0.7      stringr_1.4.0    generics_0.1.1
[17] vctrs_0.3.8       grid_3.6.3       tidyselect_1.1.1 glue_1.6.0
[21] R6_2.5.1          rstatix_0.7.0    fansi_1.0.0      carData_3.0-5
[25] car_3.0-12        tidyr_1.1.4      purrr_0.3.4      magrittr_2.0.1
[29] codetools_0.2-18 backports_1.4.1  scales_1.1.1     ellipsis_0.3.2
[33] abind_1.4-5       assertthat_0.2.1 colorspace_2.0-2 ggsignif_0.6.3
[37] utf8_1.2.2        stringi_1.7.6    munsell_0.5.0    broom_0.7.11
[41] crayon_1.4.2
```

GitHub

For reading more easily, we recommend opening the file *Data supplements.Rproj* with R-Studio, which lets the user have the correct working path set up automatically. Other files can be opened directly by double-clicking on them, or via the `Files` tab in R-Studio.
Most of the code can be folded/collapsed into sections easily by clicking in the menu `Edit->Collapse All Output`.
Unfolding can be done by clicking either on the arrows to the left of the folded sections, or in the menu `Edit->Expand All Output`.

Directory structure

For readability, folders are displayed with a dash at the end. The folder code will be at the section *code/*.

code/

Files required to compute the main results of the article: acceleration factors and coverage probabilities.

Folder on GitHub: https://github.com/timothee-bacri/HMM_with_TMB/blob/main/code

code/linreg.cpp

Specifies the function computing the negative log-likelihood of a linear regression in `C++`. Heavily inspired by https://github.com/kaskr/adcomp/blob/master/tmb_examples/linreg.cpp.

File on GitHub: https://github.com/timothee-bacri/HMM_with_TMB/blob/main/code/linreg.cpp
File: https://github.com/timothee-bacri/HMM_with_TMB/raw/main/code/linreg.cpp

code/linreg_extended.cpp

Similar to *code/linreg.cpp*, with some more complex code added to serve as an example. Used to compute negative log-likelihoods in `R` with `TMB`.

File on GitHub: https://github.com/timothee-bacri/HMM_with_TMB/blob/main/code/linreg_extende d.cpp
File: https://github.com/timothee-bacri/HMM_with_TMB/raw/main/code/linreg_extended.cpp

code/main.R

Used to run procedures related to timing and comparisons presented in the article. By default, it will load results instead.

The first chunk of code it runs can be found in the file *code/setup_parameters.R*.
Afterwards, either computations are run and their results are stored in data/, or results are loaded to the current environment. The computations are written in *code/poi_hmm_tinn.R*, *code/poi_hmm_lamb.R*, *code/poi_hmm_simu1.R*, *code/poi_hmm_simu2.R*, and *code/poi_hmm_simu3.R*.

NOTE: Our scripts were tested on a workstation with 4 Intel(R) Xeon(R) Gold 6134 processors (3.7 GHz) running under the Linux distribution Ubuntu 18.04.6 LTS (Bionic Beaver) with 384 GB, and took about a week of computing time. More details at individual files.

File on GitHub: https://github.com/timothee-bacri/HMM_with_TMB/blob/main/code/main.R
File: https://github.com/timothee-bacri/HMM_with_TMB/raw/main/code/main.R

code/mvnorm_hmm.cpp

Specifies the function computing the negative log-likelihood of a m-state multivariate Gaussian Hidden Markov Model in `C++`.

File on GitHub: https://github.com/timothee-bacri/HMM_with_TMB/blob/main/code/mvnorm_hmm.cpp
File: https://github.com/timothee-bacri/HMM_with_TMB/raw/main/code/mvnorm_hmm.cpp

code/norm_hmm.cpp

Specifies the function computing the negative log-likelihood of a m-state univariate Gaussian Hidden Markov Model in `C++`.

File on GitHub: https://github.com/timothee-bacri/HMM_with_TMB/blob/main/code/norm_hmm.cpp
File: https://github.com/timothee-bacri/HMM_with_TMB/raw/main/code/norm_hmm.cpp

code/packages.R

Automatically install/load necessary packages for running *code/main.R* with all simulations.

File on GitHub: https://github.com/timothee-bacri/HMM_with_TMB/blob/main/code/packages.R
File: https://github.com/timothee-bacri/HMM_with_TMB/raw/main/code/packages.R

code/poi_hmm.cpp

Specifies the function computing the negative log-likelihood of a m-state Poisson Hidden Markov Model in `C++`.

File on GitHub: https://github.com/timothee-bacri/HMM_with_TMB/blob/main/code/poi_hmm.cpp
File: https://github.com/timothee-bacri/HMM_with_TMB/raw/main/code/poi_hmm.cpp

code/poi_hmm_lamb.R

File on GitHub: https://github.com/timothee-bacri/HMM_with_TMB/blob/main/code/poi_hmm_lamb.R
File: https://github.com/timothee-bacri/HMM_with_TMB/raw/main/code/poi_hmm_lamb.R

On our machine, this file took approximately 2h to compute.

code/poi_hmm_simu1.R

File on GitHub: https://github.com/timothee-bacri/HMM_with_TMB/blob/main/code/poi_hmm_simu1.R
File: https://github.com/timothee-bacri/HMM_with_TMB/raw/main/code/poi_hmm_simu1.R

On our machine, this file took approximately 3h30 to compute.

code/poi_hmm_simu2.R

File on GitHub: https://github.com/timothee-bacri/HMM_with_TMB/blob/main/code/poi_hmm_simu2.R
File: https://github.com/timothee-bacri/HMM_with_TMB/raw/main/code/poi_hmm_simu2.R

On our machine, this file took approximately 23h to compute.

code/poi_hmm_simu3.R

File on GitHub: https://github.com/timothee-bacri/HMM_with_TMB/blob/main/code/poi_hmm_simu3.R
File: https://github.com/timothee-bacri/HMM_with_TMB/raw/main/code/poi_hmm_simu3.R

On our machine, this file took approximately 26h30 to compute.

code/poi_hmm_simu4.R

Unlike in the other simulations, the coverage samples do not ensure that profile CIs converge.
File on GitHub: https://github.com/timothee-bacri/HMM_with_TMB/blob/main/code/poi_hmm_simu3.R
File: https://github.com/timothee-bacri/HMM_with_TMB/raw/main/code/poi_hmm_simu3.R

On our machine, this file took approximately 63h to compute.

code/poi_hmm_tinn.R

Most of their code is common:

- `Parameters and covariates` defines parameters and covariates in natural form. The simulation files generate data there.
- `Parameters & covariates for TMB` transforms parameters and covariates to their working form.
- `Estimation` computes estimates with and without TMB for further comparison. The estimates are stored in the data-frames `conf_int_*`.
- `Creating variables for the CIs` creates necessary variables for to compute confidence intervals (CIs).
- `Benchmarks` uses the `microbenchmark` (Mersmann 2021) package to accurately time the TMB_0 TMB_G TMB_H and TMB_{GH} procedures. Their times are stored in the data-frames `estim_benchmarks_df_*`
- `Profiling the likelihood` uses the `TMB` package's `tmbprofile` function to determine a profile of the likelihood, then determines a CI based on it for the corresponding working parameter. Eventually, CIs are derived when possible for the natural parameters $\hat{\boldsymbol{\lambda}}$ and $\hat{\boldsymbol{\Gamma}}$. The CIs are stored in the data-frames `conf_int_*`.
- `Bootstrap` derives a parametric bootstrap CI from the dataset, while checking that the generated data can be estimated without errors, after which we apply our label switching function to ensure that estimates of a parameter are only aggregated with estimates of the same parameter. The CIs are stored in the data-frames `conf_int_*`.
- `TMB confidence intervals` computes CIs based on `TMB`-derived standard errors. The CIs are stored in the data-frames `conf_int_*`.
- `Coverage probabilities of the 3 CI methods` simulates coverage samples based on true values when available or on estimates otherwise, then derives CIs using the three CI generation methods described above. The coverage probabilities are stored in the data-frames `conf_int_*`.
- `Fixes` uses the label-switching algorithm on the estimates in the `conf_int_*` variable to ensure they are correctly ordered, and then reorders $\hat{\boldsymbol{\Gamma}}$ in the `conf_int_*` variable to a row-wise order instead of the default column-wise order. In short this applies two fixes on the `conf_int_*` variable to make it easier to read.
  At the end, we reorder the profile likelihood based CIs in case they are not in ascending order.

A unique randomness seed is set in each of these four files before all time-consuming tasks involving some randomness. *code/setup_parameters.R* defines the random number generator's version.

code/poi_hmm_tinn.R contains an extra section: `Benchmark the same dataset many times to check the benchmark durations has low variance`. As written in its title, its role is to time estimation with and without `TMB`. However, unlike in the section `Benchmarks`, estimation is done on the same dataset everytime. If everything goes well, the times should have a negligible variance. This allows us to check if normal background activity on the computer (e.g. the user opening a window or moving the mouse) affects estimation time in any noticeable way. If it affects estimation noticeably, then we would have to apply much stricter control on background processes in order to reliably compare estimation speeds with different optimizers. Luckily, a low variance was observed, making the acceleration evidenced in this project significant.

NOTE: to be faster, some code is parallelized:

- In *code/poi_hmm_simu1.R*, bootstrapping is parallelized both in `Bootstrap` (accelerated from 83 seconds per sample to 29 seconds) and in `Coverage probabilities of the 3 CI methods` (likelihood profiling was slower with parallelization, likely due to loading `TMB` each time and the procedure being already fairly quick: from 2.8 seconds in total without to 3.5 seconds in total with).
- In *code/poi_hmm_simu2.R*, bootstrapping and likelihood profiling are both parallelized in `Bootstrap` (accelerated from 64 seconds per bootstrap sample to 21), `Profiling the likelihood`, and in `Coverage probabilities of the 3 CI methods`.
- *code/poi_hmm_simu3.R* and *code/poi_hmm_simu4.R* benefit from the same parallelization as *code/poi_hmm_simu2.R*.

Parallelization is handled with the `foreach` (Revolution Analytics and Weston, n.d.) package.

File on GitHub: https://github.com/timothee-bacri/HMM_with_TMB/blob/main/code/poi_hmm_tinn.R
File: https://github.com/timothee-bacri/HMM_with_TMB/raw/main/code/poi_hmm_tinn.R

code/setup_parameters.R

Sets up multiple variables either to define some global constants or to store useful results for easier maintenance, and compiles *code/linreg.cpp* and *code/poi_hmm.cpp*.

File on GitHub: https://github.com/timothee-bacri/HMM_with_TMB/blob/main/code/setup_parameters.R
File: https://github.com/timothee-bacri/HMM_with_TMB/raw/main/code/setup_parameters.R

data/

Files containing both datasets and the computational results. *code/main.R* stores important results in this folder, and only loads them by default.

Speed results are available after some post-processing that we leave to the reader.

Folder on GitHub: https://github.com/timothee-bacri/HMM_with_TMB/blob/main/data

data/fetal-lamb.RData

Contains `lamb`: an integer vector of with 240 data from Leroux and Puterman (1992).

File on GitHub: https://github.com/timothee-bacri/HMM_with_TMB/blob/main/data/fetal-lamb.RData
File: https://github.com/timothee-bacri/HMM_with_TMB/raw/main/data/fetal-lamb.RData

data/results_lamb.RData

File on GitHub: https://github.com/timothee-bacri/HMM_with_TMB/blob/main/data/results_lamb.R
Data
File: https://github.com/timothee-bacri/HMM_with_TMB/raw/main/data/results_lamb.RData

data/results_simu1.RData

File on GitHub: https://github.com/timothee-bacri/HMM_with_TMB/blob/main/data/results_simu1.
RData
File: https://github.com/timothee-bacri/HMM_with_TMB/raw/main/data/results_simu1.RData

data/results_simu2.RData

File on GitHub: https://github.com/timothee-bacri/HMM_with_TMB/blob/main/data/results_simu2.
RData
File: https://github.com/timothee-bacri/HMM_with_TMB/raw/main/data/results_simu2.RData

data/results_simu3.RData

File on GitHub: https://github.com/timothee-bacri/HMM_with_TMB/blob/main/data/results_simu3.
RData
File: https://github.com/timothee-bacri/HMM_with_TMB/raw/main/data/results_simu3.RData

data/results_simu4.RData

File on GitHub: https://github.com/timothee-bacri/HMM_with_TMB/blob/main/data/results_simu4.
RData
File: https://github.com/timothee-bacri/HMM_with_TMB/raw/main/data/results_simu4.RData

data/results_tinn.RData

Contains results from computations and timings run in *code/main.R*.

File on GitHub: https://github.com/timothee-bacri/HMM_with_TMB/blob/main/data/results_tinn.R
Data
File: https://github.com/timothee-bacri/HMM_with_TMB/raw/main/data/results_tinn.RData

data/tinnitus.RData

Tinnitus data collected with the "Track Your Tinnitus" (TYT) mobile application on 87 successive days, provided by the University of Regensburg and European School for Interdisciplinary Tinnitus Research (ESIT), of which a detailed description is presented in Pryss, Reichert, Langguth, et al. (2015) and Pryss, Reichert, Herrmann, et al. (2015).

A plot is available in Figure 4.1 at https://timothee-bacri.github.io/HMM_with_TMB/using-tmb.html#f
ig:4tinnitus-fig.

File on GitHub: https://github.com/timothee-bacri/HMM_with_TMB/blob/main/data/tinnitus.RData
File: https://github.com/timothee-bacri/HMM_with_TMB/raw/main/data/tinnitus.RData

functions/

Utility functions used both in `C++` files and in `R` files.

Folder on GitHub: https://github.com/timothee-bacri/HMM_with_TMB/blob/main/functions

functions/mvnorm_utils.cpp

Utility functions used in [Multivariate Gaussian HMM] to transform working parameters to their natural form, and to compute the hidden Markov chain's stationary distribution when needed.

File on GitHub: https://github.com/timothee-bacri/HMM_with_TMB/blob/main/functions/mvnorm_utils.cpp
File: https://github.com/timothee-bacri/HMM_with_TMB/raw/main/functions/mvnorm_utils.cpp

functions/mvnorm_utils.R

Useful functions to estimate multivariate Gaussian HMMs with and without `TMB` and perform various pre-processing and post-processing.

File on GitHub: https://github.com/timothee-bacri/HMM_with_TMB/blob/main/functions/mvnorm_utils.R
File: https://github.com/timothee-bacri/HMM_with_TMB/raw/main/functions/mvnorm_utils.R

functions/norm_utils.cpp

Utility functions used in [Gaussian HMM] to transform working parameters to their natural form, and to compute the hidden Markov chain's stationary distribution when needed.

File on GitHub: https://github.com/timothee-bacri/HMM_with_TMB/blob/main/functions/norm_utils.cpp
File: https://github.com/timothee-bacri/HMM_with_TMB/raw/main/functions/norm_utils.cpp

functions/norm_utils.R

Useful functions to estimate Gaussian HMMs with and without `TMB` and perform various pre-processing and post-processing.

File on GitHub: https://github.com/timothee-bacri/HMM_with_TMB/blob/main/functions/norm_utils.R
File: https://github.com/timothee-bacri/HMM_with_TMB/raw/main/functions/norm_utils.R

functions/utils.cpp

Utility functions used in *code/poi_hmm.cpp* to transform working parameters to their natural form, and to compute the hidden Markov chain's stationary distribution when needed.

File on GitHub: https://github.com/timothee-bacri/HMM_with_TMB/blob/main/functions/utils.cpp
File: https://github.com/timothee-bacri/HMM_with_TMB/raw/main/functions/utils.cpp

functions/utils.R

Useful functions to estimate Poisson HMMs with and without `TMB` and perform various pre-processing and post-processing.

File on GitHub: https://github.com/timothee-bacri/HMM_with_TMB/blob/main/functions/utils.R
File: https://github.com/timothee-bacri/HMM_with_TMB/raw/main/functions/utils.R

functions/utils.R-explanation.R

Description of the function parameters and return objects defined in *functions/utils.R*.
The other utility files are adapted to the univariate and multivariate Gaussian setting but otherwise have identical functions.

Note that the article focuses on Poisson HMMs. Hence, some utility functions in R present for the Poisson HMM are not adapted to the Gaussian HMMs.

File on GitHub: https://github.com/timothee-bacri/HMM_with_TMB/blob/main/functions/utils.R-explanation.R
File: https://github.com/timothee-bacri/HMM_with_TMB/raw/main/functions/utils.R-explanation.R

functions/utils_linreg_extended.cpp

Contains the useless example function.

File on GitHub: https://github.com/timothee-bacri/HMM_with_TMB/blob/main/functions/utils_linreg_extended.cpp
File: https://github.com/timothee-bacri/HMM_with_TMB/raw/main/functions/utils_linreg_extended.cpp

Leroux, Brian G., and Martin L. Puterman. 1992. "Maximum-Penalized-Likelihood Estimation for Independent and Markov- Dependent Mixture Models." *Biometrics* 48 (2): 545–58. https://doi.org/10.2307/2532308.

Mersmann, Olaf. 2021. *Microbenchmark: Accurate Timing Functions*. https://github.com/joshuaulrich/microbenchmark/.

Pryss, R., M. Reichert, J. Herrmann, B. Langguth, and W. Schlee. 2015. "Mobile Crowd Sensing in Clinical and Psychological Trials – A Case Study." In *2015 IEEE 28th International Symposium on Computer-Based Medical Systems*, 23–24. https://doi.org/10.1109/CBMS.2015.26.

Pryss, R., M. Reichert, B. Langguth, and W. Schlee. 2015. "Mobile Crowd Sensing Services for Tinnitus Assessment, Therapy, and Research." In *2015 IEEE International Conference on Mobile Services*, 352–59. https://doi.org/10.1109/MobServ.2015.55.

Revolution Analytics, and Steve Weston. n.d. *Foreach: Provides Foreach Looping Construct*.
